# Achieving tuberculosis elimination in Canada and the USA: giving equal weight to domestic and international efforts

**DOI:** 10.1186/s44263-024-00115-9

**Published:** 2024-12-12

**Authors:** Namrata Rana, James C. Johnston, Kevin Schwartzman, Olivia Oxlade, Pedro G. Suarez, Michel Gasana, Megan Murray, Grania Brigden, Jonathon R. Campbell

**Affiliations:** 1 Research Institute of the McGill University Health Centre, Montreal, Canada; 2 Department of Medicine, University of British Columbia, Vancouver, Canada; 3 Provincial TB Services, BC Centre for Disease Control, Vancouver, Canada; 4 Department of Medicine, McGill University, Montreal, Canada; 5 McGill International TB Centre, Montreal, Canada; 6 Respiratory Epidemiology and Clinical Research Unit, Centre for Outcomes Research and Evaluation, Research Institute of the McGill University Health Centre, Montreal, Canada; 7 Management Sciences for Health, Arlington, VA USA; 8 Tuberculosis and Other Respiratory Diseases Division, Institute of HIV/AIDS Disease Prevention and Control, Rwanda, Biomedical Center , Kigali, Rwanda; 9 Harvard T.H. Chan School of Public Health, Boston, MA USA; 10 Harvard Medical School, Boston, USA; 11 The Global Fund to Fights AIDS, Tuberculosis and Malaria, Geneva, Switzerland; 12 Department of Medicine and Department of Global and Public Health, McGill University, Montreal, Canada

**Keywords:** Tuberculosis, North America, Elimination, Latent tuberculosis infection, Collaborative action

## Abstract

A major contributor to the tuberculosis burden in the United States (US) and Canada is the progression of tuberculosis infection acquired before immigration among persons born outside the US and Canada. Domestic interventions against tuberculosis, such as those associated with tuberculosis infection testing and treatment, while critical, are alone insufficient to address tuberculosis and achieve elimination. To hasten tuberculosis elimination in North America, coupling domestic efforts with consistent funding and multifaceted support for tuberculosis detection, treatment, and prevention worldwide is necessary. These efforts will reduce tuberculosis transmission and the prevalence of tuberculosis infection in an increasingly globalized world. We discuss the epidemiologic and economic rationale for this approach, as well as current efforts and potential strategies. We further place in context benchmark tuberculosis programs that have used international funding to achieve a sustained decline in tuberculosis incidence, as exemplars for the importance of such funding to international progress towards elimination. We conclude by providing suggestions for future pathways toward sustainable programs. Following the substantial global and local response to COVID-19, we call for the same intensity to eliminate this millennia-old disease.

## The current state of TB in the United States and Canada

The World Health Organization (WHO) has called for low-incidence countries to eliminate tuberculosis (TB) by 2050—defined as < 1 case per million population annually [1]. The United States (US) and Canada will need to attain annual reductions in TB incidence of over 10% to achieve elimination by 2050. Yet, over the last decade, both countries fell far short of this mark. In the US, after a rebound in TB incidence after COVID-19, TB incidence has remained unchanged at 29 cases per million in both 2014 and 2023 [2], while in Canada, incidence *increased* 0.8% annually from 47 to 51 cases per million between 2013 and 2022 [3]. This underscores the need for new approaches to overcome existing TB elimination challenges. In this perspective, we focus on the American and Canadian contexts, although the issues discussed are also relevant to other high-income, immigrant-receiving countries such as Australia, New Zealand, Japan, and those in Western Europe.

Since the development of 1989’s Strategic Plan to Eliminate TB in the United States, concerted efforts to reduce TB have led to a decades-long downward trend in TB incidence in the US [2, 4]. Despite these reductions, a rise in TB incidence among non-US-born persons was recently reported [5]. The Canadian government has also made commitments to reduce the incidence of TB disease in Canada. Following decades of discriminatory and ineffective practices to address the TB epidemic among Inuit communities, a plan was developed to eliminate TB [6, 7]. Yet so far, the number of Inuit people affected by TB disease remains very high and may even be increasing [8]. Similar targeted commitments are absent for other populations disproportionately impacted by TB, such as people born outside Canada, and TB elimination targets continue to be unmet [7].

People born outside Canada and the US (i.e., foreign-born residents), bear the largest TB burden in both countries (> 70%), and therefore TB prevention in this group must be a priority if elimination is to ever be considered, let alone achieved [2, 7]. The annual incidence of TB in these populations is around 150 per million, staggeringly high compared with the overall TB incidence rate for non-Indigenous Canadian-born and US-born persons at < 10 per million [2, 3]. Approximately 85% of TB disease among non-US and non-Canadian-born persons is due to the progression of TB infection acquired before immigration (i.e., before arriving in Canada or the US) [2, 3]. A range of factors impact the acquisition of TB infection and its progression to TB disease in this group, including access to testing and treatment, comorbidities that increase vulnerability, and other disparities unique to foreign-born residents [2, 3, 9].

For individuals immigrating to the US and Canada, a pre-immigration medical exam (IME) aims to identify those with infectious TB disease. All permanent resident applicants and refugees are screened, while selective screening exists for temporary students and workers depending on their length of stay and country of origin [10, 11]. The IME is also used to trigger referrals for post-immigration surveillance of people at higher risk of future TB due to radiographic abnormalities or previous TB history or exposure—approximately 2–3% of those screened [12, 13]. These post-immigration surveillance efforts are extremely effective at identifying people at high risk for TB disease as they identify 10% of people who will eventually develop TB disease [14] by only screening 2–3% of new residents [12, 13]. However, this means its overall contribution to TB elimination is limited.

Both Canada and the US also test certain groups for TB infection using an interferon-gamma release assay or tuberculin skin test as part of the IME [10, 11]. In Canada, TB infection testing is required only for those with specific risk factors for TB disease, which amounts to fewer than 1% of new permanent residents [10, 15]. Historically, the US had included TB infection testing in their IME for all children 2–15 years of age immigrating from a country with a TB incidence ≥ 20 per 100,000; however, in 2024 this has been expanded to all children and adults at least 2 years of age [11]. But for both Canadian and American programs, those with a positive TB infection test do not have any requirement to complete TB preventive treatment (TPT) prior to immigration, and treatment is primarily administered post-arrival [10, 11]. While the number of individuals receiving such treatment post-arrival is uncertain, expanded TB infection testing programs will require a proportional expansion in treatment programs, and there will be a growing need to bolster domestic capacity to provide TPT to the many individuals expected to test positive.

Though the IME is performed during the highest TB risk period among new immigrants, there are limitations. First, not all people coming to the US or Canada, such as short-term temporary workers and students, need to receive the IME. These populations may have an overall lower risk of TB, but by virtue of their large numbers, account for an appreciable proportion of TB that occurs [8]. Second, current TB infection diagnostic tests have imperfect sensitivity and specificity, and many individuals with no risk factors who test positive with such tests have < 1% lifetime risk of developing TB disease, given most were infected many years prior [16–18]. This prognostic limitation is only further exacerbated with time since arrival. Though the risk for TB falls with time since arrival, the cumulative risk of TB disease more than 5 years after arrival remains larger than the cumulative risk in the first 5 years. This dispersed low risk presents a challenge for TB infection screening and treatment programs among established residents as only a minority are at clearly elevated risk of TB disease due to identifiable risk factors [14]. Third, current TPT regimens may cause adverse events serious enough to stop treatment in 2–4% of individuals [19], and thus the individual net benefit of TPT for many is uncertain. Finally, there are weak cascades of care during follow-up post-migration through to completion of TPT, limiting overall impact [20, 21]. Key barriers along the cascade of care include the need for multiple visits to initiate treatment, failure to recommend TPT by providers, decline of TPT by patients, tolerability and duration of current TPT regimens, and the inconvenience of TPT, to name a few. While there are promising interventions to address these barriers, they will need to be adapted to local contexts [22].

Broad-based TB infection programs and their impact on progress to TB elimination have been heavily studied through modeling exercises and economic evaluations [23–25]. In all evaluations, the health system costs associated with these expanded TB infection testing and treatment programs have been found to be significant [24–26]. Further, while cost-effective in certain populations when applied at or after immigration [27], broad-based expanded screening programs have seldom been found cost-effective [24, 28, 29]. Though these programs may have a significant impact on TB epidemiology, they have consistently been found to be insufficient to eliminate TB [24, 28, 29]. Thus, while we must continue evidence-based domestic strategies (e.g., targeted screening and treatment of tuberculosis infection among high-risk populations, contact investigation, infection control) and bring to scale new domestic strategies that are effective and cost-effective at reducing TB incidence among foreign-born residents, we must recognize domestic strategies alone will not eliminate TB within our borders and may come at substantial cost. To move towards TB elimination in Canada and the US, we must not only manage TB domestically but also further emphasize a global approach to tackling TB.

## The case for a global approach to TB prevention

Real-world and modeling evidence support approaches aimed at accelerating TB elimination by also investing in TB prevention and care outside the US and Canada. First, TB incidence rates in the US and Canada among those migrating to these countries are highly correlated with TB incidence rates in their countries of origin [30–32]. Second, in both the US and Canada, 25–30% of TB disease in foreign-born residents arises among those who arrived in the previous five years [33, 34]. Therefore, by investing in global TB elimination, potential direct benefits may accrue over a relatively short time horizon. With decreased global TB transmission and frequency of exposure, people migrating to Canada and the US will have a lower prevalence of TB infection and be less likely to develop TB disease. Modeling studies also illustrate the cost-saving benefits of global investments. In the early 2000s, it was predicted that targeted investment by the US in Mexican, Dominican, and Haitian National TB programs would be net cost saving to the US health system through reductions in TB-related costs in migrants entering the US [35–37]. More recent modeling suggests the US could avert $12 billion in domestic healthcare spending and productivity loss if currently available technologies for TB were scaled up internationally [23]. This study also found that while domestic efforts against TB were impactful, they alone were insufficient to eliminate TB in the US. Indeed, only a combination of domestic and international efforts could eliminate TB in the US, further emphasizing the importance of broadening our global focus on TB.

Global public health efforts driven by the US and Canada have seen major successes and are implemented through several different initiatives. For example, TB REACH, established in 2009, is an initiative that provides project-based funding for interventions with the potential to advance TB detection and treatment. Primarily supported by Global Affairs Canada with funding from The Bill and Melinda Gates Foundation and USAID, this initiative has funded over 300 projects across 56 countries to date. The interventions supported also provide important data and information to support domestic scale-up and/or implementation of innovative technologies and approaches. Another example is the Global Drug Facility (GDF) which is a bulk purchaser of quality-assured TB medications and diagnostics [38]. Canada was a founding donor of the GDF and USAID is now the largest ongoing contributor [39, 40]. Within 4 years of its establishment, GDF became a leading initiative against TB that continues to be strongly funded [41].

In the case of the HIV epidemic, the US President’s Emergency Plan for AIDS Relief (PEPFAR), significantly changed the landscape of HIV prevention, treatment, and care since its inception in 2003 by overcoming funding challenges and competing government interests. The 20-year effort targeting HIV/AIDS epidemics globally has saved nearly 25 million lives as annual US funding also grew from US$1.9 billion to US$6.9 billion over this period [42]. These investments have had an impact beyond HIV as well. PEPFAR-funded HIV-testing laboratories were expanded to integrate COVID-19 testing and analysis, while enhancements made to PEPFAR-supported health information systems allowed countries to rapidly adapt their COVID-19 response [43]. PEPFAR’s initiatives also supported TPT implementation [44]. In 2018, the United Nations High-Level Meeting (UNHLM) on TB called on governments to provide TPT to 30 million people living with HIV (PLHIV) and household contacts (HHC). The global sub-target of providing TPT to 6 million PLHIV was far exceeded between 2018 and 2022 with 11.3 million PLHIV receiving TPT [45], a large proportion of whom received TPT through PEPFAR-funded programs [46]. In contrast, efforts to meet the global sub-target of providing TPT to 24 million HHC fell far short, as only 4.2 million HHC received TPT [45]. This is likely to improve in the coming years as USAID has pledged to increase funding for TPT through the GDF [47].

When reflecting on the successes of PEPFAR and how similar efforts in TB could be launched, it is important to acknowledge the US$110 billion investment required to develop and implement this complex initiative [42]. While financing is not the only barrier to eliminating TB globally, much of the success in addressing the HIV epidemic in PEPFAR-supported countries clearly came from sustained global funding—which will be equally essential to eliminate TB. Similarly, TB REACH has enabled several innovators and grassroots organizations to test new technologies and approaches to TB care, such as active case-finding approaches, digital treatment support, and shorter treatments, with an emphasis on locally owned and led projects [48]. However, to support countries to sustainably integrate and scale up these innovations within their healthcare systems, there is a need for long-term financial support.

Beyond these domestic programs, the US and Canada also support international organizations. The Global Fund to Fight AIDS, Tuberculosis, and Malaria, established in 2002, is one of the largest international organizations to provide financial support to end TB—contributing 76% of all international financing for TB and supporting 1.5 million people exposed to TB with TPT in 2022. The US has consistently been the Global Fund’s largest donor contributing US$24 billion to date with Canada being the seventh largest donor contributing CAD$4.5 billion to date [49]. Despite these efforts, a considerable funding gap remains between what is requested by countries and what can be provided by the Global Fund. In the first three windows of the latest funding round for Global Fund grants, there was a funding gap of US$1.2 billion, with the main gaps in TB diagnostics, treatment, and care—particularly commodities [50].

## The impact of international support on TB prevention and care: country case studies

International support has been critical to the successes seen in reducing the TB burden in some high-incidence countries. Rwanda and Peru are two high-TB incidence countries—with high and low HIV prevalence, respectively—with TB programs that have each benefitted from substantial TB-specific international funding and in parallel, have experienced significantly reduced TB incidence, with sustained declines [51, 52]. The TB responses in Rwanda and Peru were multifaceted but underfunding had been a consistent issue. With sufficient funding, health systems were strengthened and better adapted to each country’s epidemiologic context. This allowed TB programs to implement tailored, effective approaches that resulted in substantial reductions in TB incidence and mortality. While these countries do not contribute a large immigrant population to North America [53, 54], their successes in the long-term reduction of TB incidence are relevant to the US, Canada, and other immigrant-receiving, low TB incidence countries. These case studies demonstrate the potential impact increased global investment in TB prevention and care could have for the US and Canada, particularly when provided to high TB incidence countries where many new immigrants emigrate.

### Rwanda case study

In the year 2000, Rwanda (population 8 million) had a generalized HIV epidemic. More than half of the 5,815 people newly diagnosed with TB were living with HIV and the annual funding for the TB program was approximately US$1 million [55]. Over the subsequent decade, annual funding for the TB program increased to US$15 million, largely from external sources (e.g., The Global Fund has provided US$77 million since 2002). The Rwandan economy expanded rapidly with the improvement of social and economic conditions post-civil war and genocide, which also led to the development of a decentralized health system and community-based health insurance. Strengthening these systems through performance-based financing further enhanced existing services while also leading to the proliferation of TB surveillance systems. This enabled data to be generated and used to enhance monitoring, evaluation, and decision-making. In 2004, TB and HIV programs began to collaborate and there was a significant increase in the number of people with TB tested for HIV and placed on antiretroviral therapy [56, 57]. More engagement with community health workers in TB prevention activities led to a quadrupling of the number of people investigated for presumptive TB [55].

These changes coupled with an expanded number of TB care facilities resulted in TB disease treatment success rates increasing from 60% to nearly 90% [55]. From a peak incidence of 112 per 100,000 population in 2006, TB incidence in Rwanda declined to 56 per 100,000 population in 2022 [45], with the TB/HIV co-infection rate halved over this same period. While challenges remain in the capacity, financing, and implementation of TB prevention and care activities, universal health coverage and health equity remain core pillars in the Rwandan health system. TB efforts excelled due to many factors, including the country’s political and social climate, but increased funding supported the expansion of services, the implementation of prevention efforts, and the sustained commitment to reducing the TB burden.

### Peru case study

In the early 1990s, Peru accounted for 15% of the TB burden in the Americas despite accounting for only 3% of the region’s population [58]. When TB was declared a public health and social emergency, funds were mobilized to the national TB program [52] by the Peruvian government, non-governmental organizations, and international donors. Over the subsequent decade, the National TB Program (NTP) made it a priority to provide social support and treatment—free of charge—to all people with TB. This involved the rapid expansion of the TB laboratory network, the implementation of a strong surveillance system, and the establishment of a consistent supply of TB medications. TB services were decentralized and integrated into primary health care with an attendant focus on engaging and scaling the health workforce to provide these services [52]. Throughout the process, socioeconomic indicators improved, and Peru actively sought partnerships with international and national agencies. These included those based in the US (USAID), Canada (through the Peru-Canada agreement), and internationally (e.g., through the Global Fund) for technical assistance, training, and equipment tailored to national- and district-level needs [52, 59]. More recently, Peru passed a Universal Health Insurance Law, which has succeeded in providing health coverage to > 80% of the population [60]. With a TB incidence of nearly 250 per 100,000 population at its peak in the 1990s, TB incidence has fallen to 151 per 100,000 in 2022 [45]. Financial support fueled expansion of TB care and prevention, although in recent years this has stalled somewhat—particularly during the COVID-19 pandemic.

## What should be done?

Since 2015, total annual global spending on TB has been US$5.8 billion—less than half of the annual US$13 billion required as estimated by WHO [35]. The majority of these expenditures reflect domestic funding programs, with approximately 20% funded by international contributions [35].

In Canada, annual international funding provided by Global Affairs Canada for “TB control” has remained stagnant, around $50 to $60 million since 2017 (approximately 50% the size of the domestic TB budget) [26, 61]. In contrast, funding in the USA for ‘Global TB’ rose from $233 million in 2013 to $406 million in 2023 [62]; this figure includes funding provided through USAID and is about five-times larger than the annual federal funding to state TB programs through the CDC TB Elimination and Laboratory Cooperative Agreement [63]. These are not small numbers but as a proportion of Canadian and American global health budgets, reflect < 5%; moreover, Global Affairs Canada will be reducing their overall budget over the next several years. Without Canada, the US, and other countries increasing international support for TB—in parallel with increases in domestic funding by all countries—global underfunding for TB will persist. Despite large pledges made by countries to the Global Fund, USAID, and other donor organizations, international investment in improving TB care remains insufficient. Failure to increase funding and address the global TB epidemic is estimated to have an economic cost of US$1 trillion by 2030 according to The Global Plan to End TB [64]. These funding shortfalls substantially impede the development and scale-up of technologies needed to accelerate TB elimination at the global and country levels.

One key example of a potentially revolutionary technology at the global level is the development of a novel vaccine. A novel TB vaccine with modest efficacy could avert over US$3 billion in TB treatment costs from 2025 to 2050 [65] and is projected to offer a substantial return on investment while also improving long-term economic growth in countries with high TB incidence [65]. Between 2018 and 2022, US$760 million was pledged to advance TB vaccines but only US$115 million was actually invested [65]. Since 2019, the US National Institutes of Health has contributed almost half of all money spent on TB vaccine research, while the Gates Foundation has contributed around a third of the total; in comparison, the Canadian Institutes of Health Research has contributed < 1% [66]. Continued commitment by the US, and strengthening commitments in Canada, to develop a novel TB vaccine should be a key priority.

At the country level, TB programs have internal financing mechanisms which can be bolstered for sustainability and expansion, while technical assistance can be shared to support TB-related activities. The US and Canada have experience in this regard and support could be targeted to high-incidence countries. Though imperfect, our armamentarium of interventions to identify, treat, and prevent TB is stronger than ever, and scaling such technologies offers an impressive return on investment [67]. Recent randomized trials have demonstrated notable benefits of case-finding activities [68] and highlighted sustainable strategies to address TPT scale-up [69]. More generally, improving private–public partnerships within the health system, decentralizing TB care, addressing underlying drivers of TB such as undernutrition and poverty, and advancing towards Universal Health Care (UHC) can improve health system access, identification of TB disease, and implementation of TPT [45].

In September 2023, a second UNHLM on TB was held to evaluate progress against the TB epidemic. As priorities were reassessed, member countries set new, ambitious targets including reaching 90% of people who develop TB disease with quality-assured diagnostics and treatment, and providing 90% of people at high risk of TB disease with TPT by 2027 [70]. Aligning with the UNHLM’s goals, PEPFAR has launched a commitment to prevent 500,000 TB-related deaths and 2 million people developing TB disease [71]. Similarly, USAID announced an investment of more than US$23 million in new funding in addition to its USAID Global Accelerator to End TB Plus package [72]—aimed, in part, at driving an inclusive TB response, including expansion of TB case finding at primary care levels, support for TB programs in conflict settings, and increased drug accessibility. Canadian efforts to support TB elimination have also been ongoing with Global Affairs Canada investing US$12.5 million in 2020 for TB research [66], while also being a continuous core funder of TB REACH [73]. Finally, the recent Global Fund replenishment earmarks approximately $800 million annually over the next 3 years for TB in recipient countries [74].

**Fig. 1:**
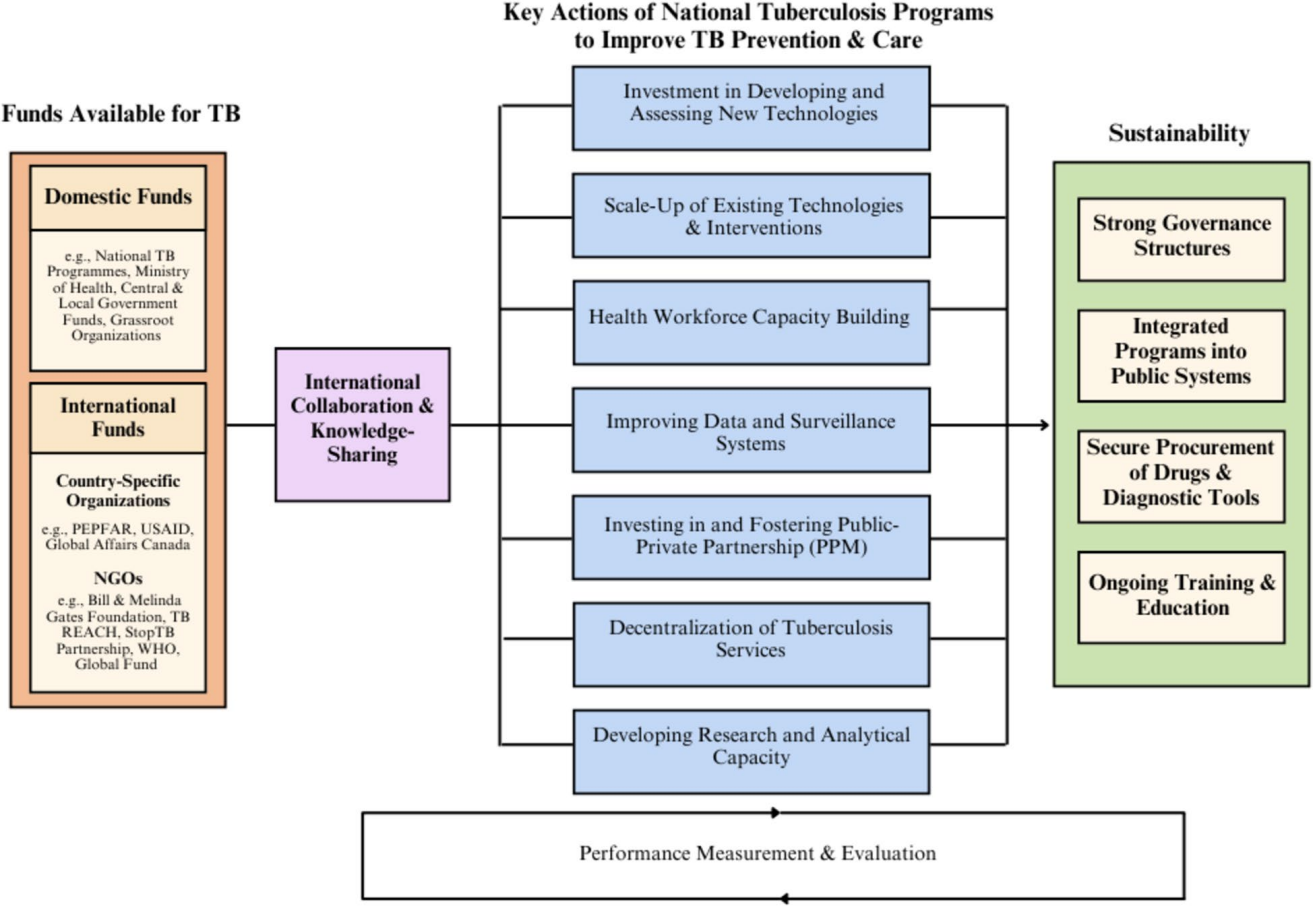
Pathway to a sustainable, global tuberculosis response

These initiatives are an excellent start, but more is needed. As well, beyond increasing funds available to diagnose, treat, and prevent TB, coordinated efforts are required, with bilateral collaboration, knowledge sharing, and sustained action to ensure lasting progress (Fig. 1). The Immigration and Refugee Health Working Group (IRHWG) is composed of Canada, US, Australia, New Zealand, and the UK and is involved in screening > 2 million overseas immigrant applicants each year. The success of these programs requires local collaboration and capacity and has led to the development of laboratory infrastructure, capacity for tuberculosis screening and treatment, and training opportunities for in-country healthcare personnel [75]. Such a working group could further be leveraged to continue building capacity, including in tuberculosis prevention. Beyond this multinational working group, in Vietnam, active, community-wide case detection programs are a product of collaboration between the University of Sydney, Woolcock Institute, and the Vietnam National Tuberculosis Program [76]. More recently, the US government has partnered with the Department of Health in the Philippines, a high-burden country, to fund a new initiative called the Support Wide-scale Interventions To Find TB (SWIF-TB) to amplify and expand TB elimination efforts [74]. The additional funding of US$21 million is comprised of US$10 million from USAID and private sector partners and a matching commitment of US$11 million by the Department of Health to support TB screening, use of TPT, and integrate TB within the care of other diseases [74, 77]. These partnerships are a great first step towards supporting country-led changes to TB prevention and care. The success of such bilateral collaborations requires ownership and political will at the country level to develop and continually fund sustainable programs, and a commitment to strengthen the health system with technological and human resources required to maintain such programs, with priorities identified by country stakeholders. Integrated performance monitoring to routinely assess these priorities will also support the success of the long-term goals of these programs while forging key collaborations at the country-level [78]. This allows donor funds, such as those from USAID provided to the Philippines, to serve as a catalyst to improve national TB elimination efforts while decision-making is retained by country leadership.

In all cases, additional funding and collaborations should prioritize health system strengthening, the development of self-sustaining infrastructure and programs, and stable internal funding mechanisms, which will promote longevity in TB efforts. The hazards of interrupted funding and lack of sustained focus on TB prevention activities were highlighted by the COVID-19 pandemic, as years of progress towards TB elimination were reversed. Similarly, challenges to TB prevention and care activities can result in substantial increases in TB incidence [79], as seen in the Canadian North [80] and previously in New York City [81].

## Conclusions

High-income, low-incidence countries share many of the same challenges in eliminating TB. Only focusing on domestic efforts to achieve elimination will predictably fail, if not coupled with an equal investment in an effective global response to the TB epidemic. We must give equal weight to domestic and international efforts to eliminate TB.

For the US and Canada, and other similar countries, forging collaborative partnerships with TB programs in higher-incidence countries likely carries significant mutual public health and economic benefits. With the 2023 UN High-Level Meeting on TB behind us, now is a good time to re-evaluate our elimination strategies. The US and Canada have committed to strengthening global health security and have pledged substantial funding to help end TB. They, and other similar countries, must double down and also recognize the benefits and potential economic efficiencies facilitated by international efforts to their own domestic healthcare systems and local efforts to eliminate TB. Without continued action and investment in global TB prevention and care, we risk undermining the progress made toward TB elimination so far. The political will and global response to COVID-19 are evidence of what can be achieved rapidly when funding, coordination, and global efforts are aligned. Supporting global TB elimination efforts with similar intensity as our domestic efforts could save millions of lives needlessly lost to a preventable and treatable disease. Conversely, without a united global commitment, we will never eliminate TB, in the US, Canada, or anywhere, and bring justice for society’s most vulnerable—those living in impoverished, marginalized, resource-constrained settings—who continue to bear the brunt of this millennia-old disease.

## Data Availability

No datasets were generated or analysed during the current study.

## References

[1] WHO. Towards TB elimination: an action framework for low-incidence countries. Geneva: World Health Organization; 2014. 25473715

[2] Williams PM. Tuberculosis — United States, 2023. MMWR Morb Mortal Wkly Rep. 2024;73:297–303. 10.15585/mmwr.mm7312a4 PMC10986816 38547024

[3] Public Health Agency of Canada. Tuberculosis in Canada: Infographic (2022). 2024.

[4] Centers for Disease Control (CDC). A strategic plan for the elimination of tuberculosis in the United States. MMWR Morb Mortal Wkly Rep. 1989;38:269–72. 2495428

[5] CDC. Tuberculosis Elimination Priorities. National Center for HIV, Viral Hepatitis, STD, and Tuberculosis Prevention. 2024. https://www.cdc.gov/nchhstp/priorities/tuberculosis-elimination.html. Accessed 9 Aug 2024.

[6] Health Canada. Office of Audit and Evaluation. Evaluation of the Public Health Agency of Canada’s Tuberculosis Activities 2015-16 to 2020-21. Health Canada and the Public Health Agency of Canada = Santé Canada et l’Agence de la santé publique du Canada; 2023.

[7] Heffernan C, Rowe BH, Long R. Engaging frontline providers: an important key to eliminating tuberculosis in Canada, and other high-income countries. Can J Public Health. 2021;112:872–6. 34515944 10.17269/s41997-021-00556-x PMC8436580

[8] Public Health Agency of Canada. Tuberculosis in Canada: 2012 to 2021 expanded report. statistics. 2024.

[9] Hayward S, Harding RM, McShane H, Tanner R. Factors influencing the higher incidence of tuberculosis among migrants and ethnic minorities in the UK. F1000Research. 2018;7:461. 30210785 10.12688/f1000research.14476.1 PMC6107974

[10] Public Health Agency of Canada. Chapter 13: Canadian tuberculosis standards 7th Edition: 2014 – tuberculosis surveillance and screening in selected high-risk populations. Canadian Tuberculosis Standard, 7th edition. 2014.

[11] CDC. Tuberculosis. Immigrant and Refugee Health. 2024. https://www.cdc.gov/immigrant-refugee-health/hcp/civil-surgeons/tuberculosis.html. Accessed 10 Aug 2024.

[12] Connecticut State Department of Public Health. Class B TB Notifications. https://portal.ct.gov/dph/tuberculosis/local-health-departments-and-tuberculosis/class-b-tb-notifications. Accessed 10 Aug 2024.

[13] Khan K, Hirji MM, Miniota J, Hu W, Wang J, Gardam M, et al. Domestic impact of tuberculosis screening among new immigrants to Ontario, Canada. CMAJ. 2015;187:E473–81. 26416993 10.1503/cmaj.150011 PMC4627893

[14] Ronald LA, Campbell JR, Rose C, Balshaw R, Romanowski K, Roth DZ, et al. Estimated Impact of World Health Organization Latent Tuberculosis Screening Guidelines in a Region With a Low Tuberculosis Incidence: Retrospective Cohort Study. Clin Infect Dis Off Publ Infect Dis Soc Am. 2019;69:2101–8. 10.1093/cid/ciz188 PMC6880326 30856258

[15] Public Health Agency of Canada. Canadian tuberculosis standards: 7th Edition. Ottawa (ON): Government of Canada; 2014.

[16] Campbell JR, Winters N, Menzies D. Absolute risk of tuberculosis among untreated populations with a positive tuberculin skin test or interferon-gamma release assay result: systematic review and meta-analysis. BMJ. 2020;368: m549. 32156698 10.1136/bmj.m549 PMC7190060

[17] Houben RMGJ, Dodd PJ. The Global Burden of Latent Tuberculosis Infection: A Re-estimation Using Mathematical Modelling. PLOS Med. 2016;13: e1002152. 27780211 10.1371/journal.pmed.1002152 PMC5079585

[18] Jordan AE, Nsengiyumva NP, Houben RMGJ, Dodd PJ, Dale KD, Trauer JM, et al. The prevalence of tuberculosis infection among foreign-born Canadians: a modelling study. CMAJ. 2023;195:E1651–9. 38081633 10.1503/cmaj.230228 PMC10718277

[19] Winters N, Belknap R, Benedetti A, Borisov A, Campbell JR, Chaisson RE, et al. Completion, safety, and efficacy of tuberculosis preventive treatment regimens containing rifampicin or rifapentine: an individual patient data network meta-analysis. Lancet Respir Med. 2023;11:782–90. 36966788 10.1016/S2213-2600(23)00096-6 PMC11068309

[20] Chan IHY, Kaushik N, Dobler CC. Post-migration follow-up of migrants identified to be at increased risk of developing tuberculosis at pre-migration screening: a systematic review and meta-analysis. Lancet Infect Dis. 2017;17:770–9. 28410979 10.1016/S1473-3099(17)30194-9

[21] Alsdurf H, Hill PC, Matteelli A, Getahun H, Menzies D. The cascade of care in diagnosis and treatment of latent tuberculosis infection: a systematic review and meta-analysis. Lancet Infect Dis. 2016;16:1269–78. 27522233 10.1016/S1473-3099(16)30216-X

[22] Barss L, Moayedi-Nia S, Campbell JR, Oxlade O, Menzies D. Interventions to reduce losses in the cascade of care for latent tuberculosis: a systematic review and meta-analysis. Int J Tuberc Lung Dis Off J Int Union Tuberc Lung Dis. 2020;24:100–9. 10.5588/ijtld.19.0185 32005312

[23] Menzies NA, Swartwood NA, Cohen T, Marks SM, Maloney SA, Chappelle C, et al. The long-term effects of domestic and international tuberculosis service improvements on tuberculosis trends within the USA: a mathematical modelling study. Lancet Public Health. 2024;9:e573–82. 39095134 10.1016/S2468-2667(24)00150-6 PMC11344642

[24] Greenaway C, Pareek M, Chakra C-NA, Walji M, Makarenko I, Alabdulkarim B, et al. The effectiveness and cost-effectiveness of screening for latent tuberculosis among migrants in the EU/EEA: a systematic review. Eurosurveillance. 2018;23:17. 29637889 10.2807/1560-7917.ES.2018.23.14.17-00543 PMC5894253

[25] Campbell JR, Sasitharan T, Marra F. A Systematic Review of Studies Evaluating the Cost Utility of Screening High-Risk Populations for Latent Tuberculosis Infection. Appl Health Econ Health Policy. 2015;13:325–40. 26129810 10.1007/s40258-015-0183-4

[26] Menzies D, Lewis M, Oxlade O. Costs for Tuberculosis Care in Canada. Can J Public Health Rev Can Santee Publique. 2008;99:391–6. 10.1007/BF03405248 PMC6976239 19009923

[27] Tasillo A, Salomon JA, Trikalinos TA, Horsburgh CR, Marks SM, Linas BP. Cost-effectiveness of Testing and Treatment for Latent Tuberculosis Infection in Residents Born Outside the United States With and Without Medical Comorbidities in a Simulation Model. JAMA Intern Med. 2017;177:1755–64. 29049814 10.1001/jamainternmed.2017.3941 PMC5808933

[28] Shedrawy J, Deogan C, Öhd JN, Hergens M-P, Bruchfeld J, Jonsson J, et al. Cost-effectiveness of the latent tuberculosis screening program for migrants in Stockholm Region. Eur J Health Econ. 2021;22:445–54. 33559787 10.1007/s10198-021-01265-5 PMC7954754

[29] Dale KD, Abayawardana MJ, McBryde ES, Trauer JM, Carvalho N. Modeling the Cost-Effectiveness of Latent Tuberculosis Screening and Treatment Strategies in Recent Migrants to a Low-Incidence Setting. Am J Epidemiol. 2022;191:255–70. 34017976 10.1093/aje/kwab150

[30] Cain KP, Haley CA, Armstrong LR, Garman KN, Wells CD, Iademarco MF, et al. Tuberculosis among foreign-born persons in the United States: achieving tuberculosis elimination. Am J Respir Crit Care Med. 2007;175:75–9. 17038659 10.1164/rccm.200608-1178OC

[31] Tsang CA, Langer AJ, Kammerer JS, Navin TR. US Tuberculosis Rates among Persons Born Outside the United States Compared with Rates in Their Countries of Birth, 2012–2016. Emerg Infect Dis. 2020;26:533–40. 32091367 10.3201/eid2603.190974 PMC7045845

[32] Ronald LA, Campbell JR, Balshaw RF, Romanowski K, Roth DZ, Marra F, et al. Demographic predictors of active tuberculosis in people migrating to British Columbia, Canada: a retrospective cohort study. CMAJ Can Med Assoc J J Assoc Medicale Can. 2018;190:E209–16. 10.1503/cmaj.170817 PMC5826706 29483329

[33] Public Health Agency of Canada. Tuberculosis in Canada - 2008–2018 Data - Tuberculosis disease in Canada - 2008–2018 Data - Open Government Portal. https://opencanada.blob.core.windows.net/opengovprod/resources/1ff8f1b6-02a8-425a-bd0b-af9495d2e53c/tb-in-canada_2008-2018_eng_march24-2022.pdf?se=2024-12-02T16%3A25%3A53Z&sp=r&sv=2019-07-07&sr=b&sig=GyvRIC3AAueewOqSlhqFWshIgUOTZnfLmOiDDq6zXt0%3D. Accessed 2 Dec 2024.

[34] CDCTB. Reported TB in the U.S., 2020- Executive Commentary. Centers for Disease Control and Prevention. 2023. https://www.cdc.gov/tb/statistics/reports/2022/Exec_Commentary.html. Accessed 12 Aug 2024.

[35] Schwartzman K, Oxlade O, Barr RG, Grimard F, Acosta I, Baez J, et al. Domestic returns from investment in the control of tuberculosis in other countries. N Engl J Med. 2005;353:1008–20. 16148286 10.1056/NEJMsa043194

[36] Dye C, Glaziou P, Floyd K, Raviglione M. Prospects for tuberculosis elimination. Annu Rev Public Health. 2013;34:271–86. 23244049 10.1146/annurev-publhealth-031912-114431

[37] Hill AN, Becerra JE, Castro KG. Modelling tuberculosis trends in the USA. Epidemiol Infect. 2012;140:1862–72. 22233605 10.1017/S095026881100286X

[38] Global Drug Facility (GDF) | Stop TB Partnership. https://www.stoptb.org/facilitate-access-to-tb-drugs-diagnostics/global-drug-facility-gdf. Accessed 13 Aug 2024.

[39] Babaley M. Global Drug Facility (GDF) 2020 updates. 2020.

[40] Global Affairs. Project profile — Global drug facility: provision of anti-tuberculosis drugs. 2017. https://w05.international.gc.ca/projectbrowser-banqueprojets/project-projet/details/m011474003. Accessed 15 Aug 2024.

[41] World Health Organization. 4 Million Treatments in 4 Years. https://www.who.int/publications/i/item/WHO-HTM-STB-2005-32. Accessed 2 Dec 2024.

[42] The U.S. President’s Emergency Plan for AIDS Relief (PEPFAR). KFF. 2023. https://www.kff.org/global-health-policy/fact-sheet/the-u-s-presidents-emergency-plan-for-aids-relief-pepfar/. Accessed 12 Aug 2024.

[43] Mirza M, Grant-Greene Y, Valles MPJS, Joseph P, Juin S, Brice S, et al. Leveraging PEPFAR-Supported Health Information Systems for COVID-19 Pandemic Response. Emerg Infect Dis. 2022;28(Suppl 1):S49-58. 36502426 10.3201/eid2813.220751 PMC9745247

[44] Boyd AT, Moore B, Shah M, Tran C, Kirking H, Cavanaugh JS, et al. Implementing TB preventive treatment within differentiated HIV service delivery models in global programs. Public Health Action. 2020;10:104–10. 33134124 10.5588/pha.20.0014 PMC7577005

[45] WHO. Global tuberculosis report 2023. 2023.

[46] CDC. Ending the global tuberculosis epidemic. Global HIV and TB. 2024. https://www.cdc.gov/global-hiv-tb/php/our-approach/combatingglobaltb.html. Accessed 13 Aug 2024.

[47] Stop TB Partnership. USAID catalytic funding available for 3HP Scale-up among Household and close contacts in high TB burden countries. https://www.stoptb.org/news/usaid-catalytic-funding-available-3hp-scale-among-household-and-close-contacts-high-tb-burden. Accessed 13 Aug 2024.

[48] Stop TB Partnership. By projects -TB REACH. TB reach projects map: innovative solutions for TB case detection. https://tbreach.org/projects/. Accessed 12 Aug 2024.

[49] The Global Fund. Results report 2023. https://www.theglobalfund.org/en/results/. Accessed 12 Aug 2024.

[50] The Global Fund. Technical review panel observatiosn report - C19RM Portfolio Optimization Wave 2. https://www.theglobalfund.org/media/13662/trp_2023-observations-c19rm_report_en.pdf. Accessed 2 Dec 2024.

[51] Rwanda Biomedical Centre. Rwanda Tuberculosis National Strategic Plan (TB NSP): July 2013-June 2018. Kigali, Rwanda; 2014.

[52] Talbot JR, Rhatigan J, Kim JY. The Peruvian national tuberculosis control program. Massachusetts: Harvard Business Publishing; 2011.

[53] Statistics Canada. Place of birth and period of immigration by gender and age: Canada, 2023. Government of Canada. https://open.canada.ca/data/en/dataset/a19c74b0-4547-476e-b193-31e540e7653e. Accessed 2 Dec 2024.

[54] U.S. Census Bureau. Place of birth for the foreign-born population in the United States. American Community Survey, ACS 5-Year Estimates Detailed Tables, Table B05006. 2022.

[55] Klinkenberg E. Epidemiological review and impact analysis of tuberculosis in Rwanda. Netherlands: KNCV; 2014.

[56] Ndagijimana A, Rugigana E, Uwizeye CB, Ntaganira J. One-stop TB-HIV services evaluation in Rwanda: comparison of the 2001–2005 and 2006–2010 cohorts. Public Health Action. 2015;5:209–13. 26767172 10.5588/pha.15.0093 PMC4682610

[57] Uwinkindi F, Nsanzimana S, Riedel DJ, Muhayimpundu R, Remera E, Gasana M, et al. Scaling Up Intensified Tuberculosis Case Finding in HIV Clinics in Rwanda. JAIDS J Acquir Immune Defic Syndr. 2014;66: e45. 24562350 10.1097/QAI.0000000000000128

[58] Suárez PG, Watt CJ, Alarcón E, Portocarrero J, Zavala D, Canales R, et al. The Dynamics of Tuberculosis in Response to 10 Years of Intensive Control Effort in Peru. J Infect Dis. 2001;184:473–8. 11471105 10.1086/322777

[59] Llanos-Zavalaga F, Poppe P, Tawfik Y, Church-Balin C. The role of communication in Peru’s fight against tuberculosis. Baltimore: Health Communication Partnership; 2004.

[60] Alarcón V, Alarcón E, Figueroa C, Mendoza-Ticona A. Tuberculosis in Peru: epidemiological situation, progress and challenges for its control. Rev Peru Med Exp Salud Pública. 2017;34:299–310. 29177392 10.17843/rpmesp.2017.342.2384

[61] Global Affairs Canada. DevData dashboard. GAC. 2021. https://www.international.gc.ca/transparency-transparence/international-assistance-report-stat-rapport-aide-internationale/dashboard-tableau-bord.aspx?lang=eng. Accessed 12 Aug 2024.

[62] KFF. Breaking down the U.S. Global health budget by program area. 2024. https://www.kff.org/global-health-policy/fact-sheet/breaking-down-the-u-s-global-health-budget-by-program-area/. Accessed 12 Aug 2024.

[63] Government of USA. Search Results Detail | Grants.gov. Tuberculosis elimination and laboratory cooperative agreement. https://www.grants.gov/search-results-detail/352484. Accessed 25 Sep 2024.

[64] Stop TB Partnership. Global Plan to End TB 2023–2030. https://omnibook.com/embedview/dc664b3a-14b4-4cc0-8042-ea8f27e902a6/page-en.html?no-ui. Accessed 2 Dec 2024.

[65] WHO. An investment case for new tuberculosis vaccines. Geneva: WHO. https://www.who.int/publications/i/item/9789240064690. Accessed 2 Dec 2024.

[66] Tomlinson C. Tuberculosis Research Funding Trends, 2005-2020. NY, USA: Published by Treatment Action Group; 2021. https://www.treatmentactiongroup.org/wp-content/uploads/2021/12/tb_funding_2021.pdf. Accessed 2 Dec 2024.

[67] Vesga JF, Mohamed MS, Shandal M, Jabbour E, Lomtadze N, Kubjane M, et al. The return on investment of scaling tuberculosis screening and preventive treatment: a modelling study in Brazil, Georgia, Kenya, and South Africa. 2024;:2024.03.12.24303930. https://www.medrxiv.org/content/10.1101/2024.03.12.24303930v1. 10.1016/S2214-109X(25)00321-3 41109257

[68] Marks GB, Nguyen NV, Nguyen PTB, Nguyen T-A, Nguyen HB, Tran KH, et al. Community-wide Screening for Tuberculosis in a High-Prevalence Setting. N Engl J Med. 2019;381:1347–57. 31577876 10.1056/NEJMoa1902129

[69] Oxlade O, Benedetti A, Adjobimey M, Alsdurf H, Anagonou S, Cook VJ, et al. Effectiveness and cost-effectiveness of a health systems intervention for latent tuberculosis infection management (ACT4): a cluster-randomised trial. Lancet Public Health. 2021;6:e272–82. 33765453 10.1016/S2468-2667(20)30261-9

[70] WHO. World leaders commit to new targets to end TB. 2023. https://www.who.int/news/item/22-09-2023-world-leaders-commit-to-new-targets-to-end-tb. Accessed 12 Aug 2024.

[71] PEPFAR launches new effort to fight TB: goal to detect two million cases and prevent 500,000 deaths. United States Department of State. https://www.state.gov/pepfar-launches-new-effort-to-fight-tb-goal-to-detect-two-million-cases-and-prevent-500000-deaths/. Accessed 5 Oct 2023.

[72] USAID Launches New Efforts in Fight Against Tuberculosis, Including Additional $23 Million in Funding | Press Release. U.S. Agency for International Development. 2023. https://www.usaid.gov/news-information/press-releases/sep-22-2023-usaid-launches-new-efforts-fight-against-tuberculosis-including-additional-23-million-funding. Accessed 5 Oct 2023.

[73] TB REACH | Stop TB Partnership. https://www.stoptb.org/accelerate-tb-innovations/tb-reach. Accessed 16 Oct 2023.

[74] The Global Fund. Allocation funding. https://www.theglobalfund.org/en/applying-for-funding/sources-of-funding/allocation-funding/. Accessed 12 Aug 2024.

[75] Douglas P, Posey DL, Zenner D, Robson J, Abubakar I, Giovinazzo G. Capacity strengthening through pre-migration tuberculosis screening programmes: IRHWG experiences. Int J Tuberc Lung Dis Off J Int Union Tuberc Lung Dis. 2017;21:737–45. 10.5588/ijtld.17.0019 PMC10461077 28633697

[76] Woolcock Institute of Medical Research. ACT 5. Woolcock Vietnam. https://www.woolcockvietnam.org/act-5. Accessed 12 Feb 2024.

[77] U. S. Embassy. U.S., Philippines announce Php1.15-Billion Partnership to fight tuberculosis. U.S. Embassy in the Philippines. 2024. https://ph.usembassy.gov/u-s-philippines-announce-php1-15-billion-partnership-to-fight-tuberculosis/. Accessed 25 Sep 2024.

[78] Heffernan C, Haworth-Brockman M, Plourde P, Wong T, Ferrara G, Long R. Chapter 15: Monitoring tuberculosis program performance. Can J Respir Crit Care Sleep Med. 2022;6:229–41.

[79] Kimbrough W, Saliba V, Dahab M, Haskew C, Checchi F. The burden of tuberculosis in crisis-affected populations: a systematic review. Lancet Infect Dis. 2012;12:950–65. 23174381 10.1016/S1473-3099(12)70225-6

[80] Dehghani K, Lan Z, Li P, Michelsen SW, Waites S, Benedetti A, et al. Determinants of tuberculosis trends in six Indigenous populations of the USA, Canada, and Greenland from 1960 to 2014: a population-based study. Lancet Public Health. 2018;3:e133–42. 29426597 10.1016/S2468-2667(18)30002-1

[81] Frieden TR, Fujiwara PI, Washko RM, Hamburg MA. Tuberculosis in New York City–turning the tide. N Engl J Med. 1995;333:229–33. 7791840 10.1056/NEJM199507273330406

